# HTLV infection in blood donors from Mato Grosso do Sul state: a closer look at HTLV screening in Brazilian blood banks

**DOI:** 10.1038/s41598-023-41875-y

**Published:** 2023-09-04

**Authors:** Carolina Amianti, Larissa Melo Bandeira, Juliana Santos Romeiro, Bibiana Rugolo Oliveira Nakao, Marli Terezinha Micharki Vavas, João Américo Domingos, Silvia Naomi de Oliveira Uehara, Ana Rita Coimbra Motta-Castro

**Affiliations:** 1 Universidade Federal de Mato Grosso do Sul, Campo Grande, MS Brazil; 2 Secretaria de Estado de Saúde de Mato Grosso do Sul, Campo Grande, MS Brazil; 3 Fiocruz Mato Grosso do Sul, Fundação Oswaldo Cruz/Ministério da Saúde, Campo Grande, Brasil

**Keywords:** Infectious-disease diagnostics, Epidemiology, HTLV

## Abstract

Human T-lymphotropic virus (HTLV) infection has a worldwide distribution and currently, more than 2.5 million individuals have been infected in Brazil. The study aimed to investigate HTLV infection prevalence among blood donors in Mato Grosso do Sul, characterizing seroepidemiological profiles of HTLV-1/2 positive individuals and evaluating the blood bank's HTLV screening system. A cross-sectional survey was conducted among blood donors from Mato Grosso do Sul state (MS)—Central Brazil, between January to December 2021. The information was obtained from databases, samples from the collection of HEMOSUL, and active searching, with the completion of laboratory analyses. 35,278 blood donors were screened for anti-HTLV-1/2 by chemiluminescence immunoassay (CMIA). Among them, 78 were initially reactive for anti-HTLV-1/2 (2.21/1000). Out of 78, 67 returned to the blood center to collect a second sample for retesting with a second screening with CMIA. After confirmation, 8 samples were indeterminate, and 8 were confirmed as positive for HTLV antibodies. New tests were performed for the 8 positive samples, and 6 were confirmed as HTLV-1 infection (0.17/1,000), one as negative, and one as indeterminate. The present study describes the low prevalence of HTLV infection in blood donors from MS and contributes to the definition of the regional infection profile. The prevalence found in this study (0.017%–0.17/1000) shows to be a much lower value than the rates reported in other states in Brazil. We highlight the need for confirmatory testing for those seropositive donors in screening assays and the need for adequate counseling and patient management for those confirmed HTLV individuals.

## Introduction

Human T-lymphotropic virus (HTLV) is a retrovirus with a tropism for T lymphocytes. HTLV-1 and HTLV-2 were identified in 1980 and 1982, respectively, besides HTLV-3 and HTLV-4 in 2005^1–4^. HTLV-1 is mostly associated with adult T-cell leukemia (ATL) and HTLV-1-associated myelopathy (HAM) but is also associated with other inflammatory diseases^5,6^. HTLV-1 has a worldwide distribution, with South Japan, sub-Saharan Africa, Caribbean Islands, Melanesia, and South America considered the most prevalent regions^7^.

Considering that blood transfusion can be a route for HTLV transmission, screening of blood donors is important to prevent it. Thereat, screening for HTLV-1/2 infection in Brazilian blood banks has become mandatory since 1993^8^. From the data obtained, several seroprevalence studies were carried out. It is estimated that 2.5 million individuals are infected with HTLV-1 in Brazil, and a heterogeneous prevalence was observed according to the region and specific populations^9^. Previous studies conducted in the state of Mato Grosso do Sul (Central Brazil) have reported rates varying from 0.1%—10.0% in specific groups, such as pregnant women^10^, indigenous population^11^, prisoners^12^, Japanese community^13,14^, men who have sex with men^15^ and remnant quilombos^16^.

This cross-sectional study aimed to describe the HTLV infection prevalence in blood donors in Mato Grosso do Sul state and the seroepidemiological profile of the positive HTLV-1/2 individuals, and also evaluate the screening system for HTLV of the blood bank.

## Results

A total of 45,751 blood donations were screened between January 2021 to December 2021, corresponding to 35,278 blood donors. The general profile of blood donor candidates based on self-report before the donation was in majority men (55.48%), with ages ranging from 16 to 69 years (average 35 years). Most donors reported being widows (47.62%), 85.03% identify as Brazilian caucasian, and most donations were realized in the capital—Campo Grande (77.7%).

**Figure 1:**
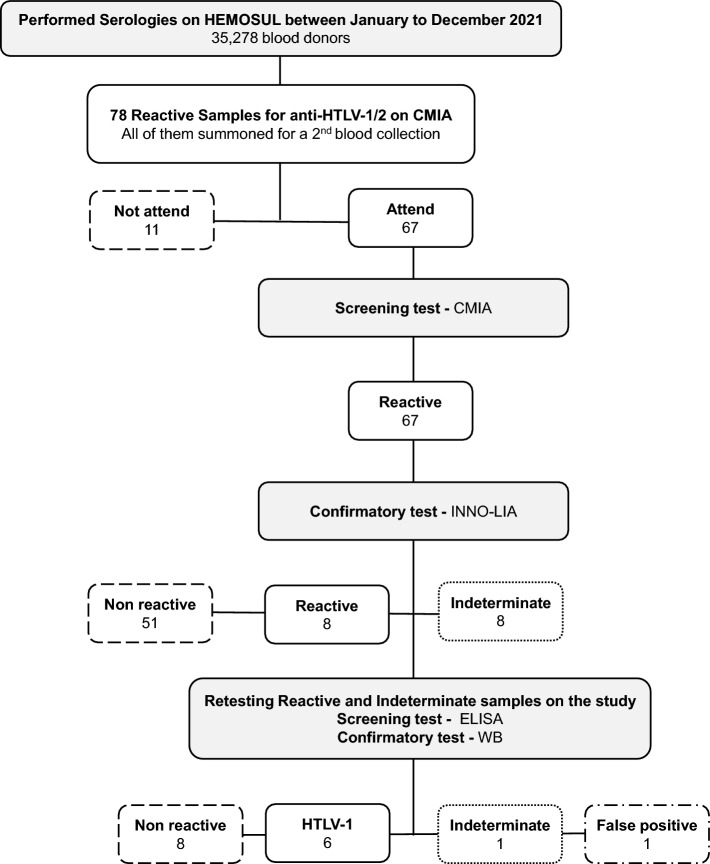
Representative flowchart about the blood tests and results performed at HEMOSUL and in the respective study.

Seventy-eight candidates were considered inapt due to being positively screened for anti-HTLV-1/2 (Fig. 1). The blood bags were discarded, the individual was blocked for further donations in both cases, and the HEMOSUL called the individuals for a second blood collection. In this group, most of them were already a donor (53/78—67.95%), and 5 showed anti-HTLV positivity in addition to other infections such as syphilis, hepatitis B (HBV), and C (HCV) infections in the screening tests. All 5 had negative results in HTLV confirmatory test. The characteristics of the eligible and inapt blood donors on the HTLV screening test are presented in Table 1.

**Table 1:** Association between the epidemiological profile of eligible and inapt blood donors based on the first HTLV screening test from MS.

Variables	Donor undetectable for HTLV (n 35,200)		Donor detectable for HTLV (n 78)	
	n	%	n	%
Sex				
Female	15,669	44.51	38	48.72
Male	19,531	55.49	40	51.28
Age				
> 25	6,538	18.57	16	20.51
25–30	6,118	17.38	14	17.95
31–40	11,188	31.78	24	30.77
< 40	11,356	32.26	24	30.77
Marital status				
Married	15,576	44.25	33	42.31
Single	26	0.07	–	–
Widower	16,767	47.63	34	43.59
Divorced	1,358	3.86	5	6.41
Others	223	0.63	1	1.28
Not informed	1,250	3.55	5	6.41
Ethnicity				
Caucasian	3,337	9.48	6	7.69
Brazilian caucasian	29,930	85.03	68	87.18
Brown	359	1.02	2	2.56
Black	1,228	3.49	1	1.28
Yellow	294	0.84	1	1.28
Indigenous	12	0.03	–	–
Not informed	40	0.11	–	–
City of donation				
Campo Grande	27,223	77.35	63	80.77
Dourados	7235	20.55	13	16.67
Corumbá	221	0.63	2	2.56
Coxim	521	1.48	–	–

All data was self-reported before donation on HEMOSUL and included in bank system, HEMOVIDA.

**Figure 2:**
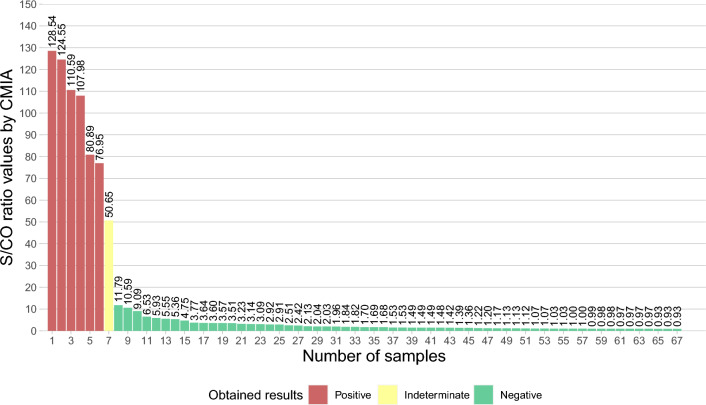
Graph representing the 67-s samples analyzed by CMIA. The columns represent the 67 samples with their respective CMIA rates results just above. In red columns are represented the 6 samples that were confirmed as positive, yellow the 1 indeterminate and green as 60 negative samples, respectively.

Of the 78 positive samples in the screening test (2.21/1000), 67 returned to the blood center to collect a second sample for retesting with a second screening with CMIA. The spread of signal/cut-off ratio (S/CO) values of the second collection samples is presented in Fig. 2. The values obtained ranged from a S/CO ratio of 0.93 to 128.54. All these samples were analyzed by the confirmatory test WB, since HEMOSUL carried out confirmatory tests until May 2022. Furthermore, all individuals with positive results received a referral to make an appointment in the public health system.

Of all 67 samples, 8 were indeterminate, and 8 were positive in confirmatory tests (HTLV-1/2 INNO-LIA) realized in HEMOSUL (Fig. 1). The indeterminate samples showed S/CO ratio values in CMIA ranging from 0.98 to 11.79, and positive from 50.65 to 128.54, except for one sample with a S/CO ratio value of 5.93 (DMS-01). Fifty-one samples were confirmed as negative, with S/CO ratio values ranging from 0.93 to 10.59 (Fig. 2).

All indeterminate samples (n = 8) from HEMOSUL were retested by our group and were confirmed negative for HTLV infection. New tests were performed for the 8 positive samples using stored blood samples from HEMOSUL or through new blood collection of the 3 individuals who agreed to participate in the study. Of these 8 samples, 6 were confirmed as HTLV-1 (0.17/1,000), one as negative (false positive) (DMS-01: S/CO ratio 5.93), and one as indeterminate (S/CO ratio 50.56).

One of the individuals who agreed to participate in the study (DMS-02) was a 46-year-old female and never donated blood before. She reported being married once when she had 2 children, and 2 cesarean surgeries were performed. Also, she reported never having a blood transfusion, having piercings or tattoos, having an accident with others’ blood, being arrested, or using drugs. She was unaware of having other IST. No information was available on the HTLV-1 status of her ex-husband. The first child was born in 2006. At that time, MS state had already been testing anti-HTLV on antenatal since 2002^10^. The youngest child (DMS-02.01) was born in 2008 and breastfed for 2 years. Both DMS-02 and DMS-02.01 had blood collected by our research group. DMS-02 was confirmed for anti-HTLV-1, and DMS-02-01 had a negative result for anti-HTLV-1/2. The participant was referred to the medical service but did not attend and was going to a city change.

The other positive (DMS-04) was already a blood donor, a 40-year-old female, and married twice. No information was available on the HTLV-1 status of the first husband with whom she had her first child in 2002, and she reported breastfeeding for 9 months. Her mother (DMS-04.02), sister (DMS-04.03), current husband (DMS-04.05), and one child of the couple (DMS-04.01) accepted to participate in the study and the serology for HTLV-1/2 were negative for all them. DMS-04 reported receiving one bag of blood transfusion in 2020 due to blood loss in childbirth. She was also reported having an accident with others’ blood, having a tattoo, being diagnosed with herpes, and sharing personal objects like pliers. DMS-04.01 was born in 2008 and was not breastfed. The couple’s youngest daughter was born in 2020 and breastfed for 8 months. She tested for anti-HTLV-1/2 in the private health system and reported that the result was negative. Participant DMS-04 was referred to the medical service. During the consultation, the doctor consulted the previous result of the last antenatal screening and reported that she already had serology positive for anti-HTLV-1, and she reported being unaware of this result.

A third blood donor candidate agreed to participate in the study (DMS-01), a 20-year-old female blood donor who had never donated blood before. The value obtained on the screening test in CMIA was a S/CO ratio value of 5.93. She reported as single in the research interview, but she reported in the blood bank questionnaire that was married. Her mother also agreed to participate in the study (DMS-01.01). Both DMS-01 and DMS-01.01 blood samples were negative HTLV-1/2 in our study. DMS-01 was referred to the medical service. She reported having rheumatologic symptoms, and other tests were ordered by the doctor.

Some inconsistencies were found comparing the answers given to HEMOSUL before the donation and to research group during the interview, such as marital status and ethnicity. Regarding the other three confirmed HTLV blood donor candidates, the information available is based on the institutional database only (Table 2). Of these 6 individuals with HTLV infection confirmed, none of them were positively screened for other infections carried out in the blood bank, such as HBV, HCV, Syphilis, and Chagas infections. The epidemiological profile of them included a predominance of male individuals (66.67%), and widows (66.67%). The mean age was 40.5 years old. The majority declared themselves to be of Brazilian caucasian ethnicity (83.33%). Regarding the geographical origin, all samples were from Campo Grande, and 50% of the anti-HTLV-1/2 positive individuals were first-time blood donors (Table 2).

**Table 2:** Profile of 6 confirmed HTLV-1 blood donor candidates from HEMOSUL.

Characteristics	DMS-02	DMS-03	DMS-04	DMS-05	DMS-06	DMS-08
Age (years)	46	38	40	37	57	42
Sex	Female	Male	Female	Male	Male	Male
Marital status	Divorced	Widower	Widower	Widower	Not informed	Widower
Ethnicity	Brazilian caucasian	Brazilian caucasian	Caucasian	Brazilian caucasian	Brazilian caucasian	Brazilian caucasian
Natality	Amambai—MS	Not informed	Campo Grande—MS	Corumbá—MS	Not informed	Campo Grande—MS
City of donation	Campo Grande	Campo Grande	Campo Grande	Campo Grande	Campo Grande	Campo Grande
First-time donation	Yes	No	No	Yes	No	Yes
Screening value on CMIA	107.98	128.54	124.55	80.89	76.95	110.59

All data were self-reported before donation on HEMOSUL and included in the bank system, HEMOVIDA.

## Discussion

Previous studies on HTLV infection prevalence in the state of Mato Grosso do Sul have focused on specific groups, such as pregnant women^10^, the indigenous population^11^, prisoners^12^, the Japanese community^13,14^, men who have sex with men^15^ and remnant quilombos^16^. The present study describes the seroepidemiological profile of blood donors from Mato Grosso do Sul state, Central Brazil. A study of blood donors from 27 Brazilian state capitals centers assessed the geographic distribution of HTLV screening prevalence rates between 1995 and 2000. It was observed a heterogeneous distribution of HTLV prevalence among the Brazilian regions, with higher rates in the Northern/Northeastern. In the state of Mato Grosso do Sul the prevalence estimated was 2.1/1,000, not considering confirmatory tests^17^. The prevalence found in the present study considering the initial screening tests (2.21/1000) indicates maintenance of the prevalence over more than 20 years between these studies. However, using confirmatory tests, the prevalence found in this study (0.017%—0.17/1000) shows to be a much lower value. Low prevalence was also observed in the pregnant population from Mato Grosso do Sul (0.13%)^10^. Other blood donor studies conducted in low prevalence regions in Brazil found rates of prevalence of 0.10%, 0.08%, 0.11%, 0.08%, 0.13%, and 0.15% in Araçatuba, Presidente Prudente, Serrana, Ribeirão Preto (São Paulo state), Manaus (Amazonas state) and in the state of Maranhão, respectively^18–20^.

The profile of eligible and inapt blood donor candidates for HTLV based on self-report is similar (Table 1). In the follow-year of monitoring the return of donors for the second collection, it was verified the difficulty that the blood bank has in contacting donor candidates since several telephone contact attempts were made without return, including sending letters to the address and telephone provided as personal contact before the first donation. In some cases, it took months to get a response for the contact, and several (11/78) did not return for the second collection.

The three positive individuals who agreed to participate in the study show some particularities. When accompanied in the medical service by the group, DMS-01 related some autoimmune symptoms, and her rheumatoid factor showed a positive result, which could have caused interference with HTLV screening, generating a false positive result in the confirmatory test realized on the blood bank. Fujirebio INNO-LIA HTLV I/II Score kit manufacturer’s instructions provide information that some infections may interfere with the analysis, as well as immunological changes such as autoimmune conditions.

DMS-02 reported during the interview that, despite the referral to schedule an appointment received from HEMOSUL, she was unable to access the health service. She had been trying to schedule for almost a year. Since forwarding had no effect, this gap shows the urgent need to organize a referral system between the blood bank managed by the reference centers since the individual already presents laboratory results, a possible diagnosis, and the clinical condition of the same must be investigated.

Participant DMS-06 was followed up throughout her entire antenatal period, but she related that she was not informed about her positive serology for anti-HTLV-1 during her last pregnancy. Her daughter was breastfed for 8 months, which could lead to mother-to-child transmission of HTLV through breastfeeding^21^. At the time, the child had 2 years old and did not present a seroconversion. The need for an improvement of maternal counseling about breastfeeding was already reported in the state^11^, demonstrating that infection patterns are not so well understood by health workers^22^ when they are expected that they should be able to properly provide counseling for positive patients about HTLV care and prevention.

Regarding inconsistencies found in the answers given to HEMOSUL and the research group, it may be that some blood donors see the blood banks as a convenient place for free testing^22^. The default in marital status found in this study may be due to the omission of risk factors, such as possibly multiple partners. Regarding ethnicity, most of them reported as Brazilian caucasian. Other studies that used data from HEMOVIDA bring the information that Brazilian caucasian include brown and mixed race^23^, and there is no clear difference between them^24^. Brazilian Institute of Geography and Statistics (IBGE) has 5 options for self-declared race/color: white, black, brown, indigenous, or yellow^25^. We suggest a standardization following the IBGE pattern applied for the blood donor candidates.

All individuals whose initial sample was anti-HTLV positive with a S/CO ratio < 11.79 was subsequently found to be HTLV negative. Although the manufacturer’s instructions of Abbott Architect rHTLV I/II assay indicate that samples with S/CO ratio ≥ 1 are considered reactive, previous studies suggest that the S/CO ratio < 4 excludes HTLV infection and the risk of blood transmission^26,27^. Then, the blood bags with S/CO < 4 may have had unnecessary disposal. Blood banks have no diagnostic purpose since the main objective is to avoid false negatives, then a high rate of false-positive results is expected in the blood bank screening tests due to low positive predictive value^28^, this implies an overestimation of the prevalence of HTLV infection if only a screening test is considered. Therefore, the algorithm of the tests influences the prevalence rate. In this study, we considered positive HTLV-confirmed result donors only. During the study period, the HEMOSUL blood bank performed confirmatory tests for HTLV on the positive samples in the screening. However, since May 2022, HEMOSUL has not performed confirmatory tests for HTLV. Furthermore, we must consider the impact of a false-positive result on the quality of life of a person without the confirmatory test to show him that he is uninfected. However, conducting confirmatory tests for HTLV is not mandatory for Brazilian blood bank services^29^.

Considering that our data showed that the HTLV-confirmed samples were only 7.69% (6/78) of the initially positive samples and that there is no specific treatment for HTLV, identifying an infected blood donor candidate represents a great opportunity to block the transmission, consequently, the associated diseases, but this requires adequate counseling and patient management. This fact highlights the importance of retesting positive blood donors and/or submitting to a confirmatory test.

We identified some limitations related to the HTLV infection prevalence in blood donors in the review literature. Most studies used initial blood bank screening data, which may result in an overestimation of prevalence due to the low cutoff value of the S/CO ratio. Another limitation of our study was the limited availability of behaviour pieces of information in the HEMOSUL database for each blood donor.

In conclusion, our findings provide additional blood donors data for the prevalence of HTLV-1/2 in Brazil and highlight the need for a closer look at HTLV infection results in Brazilian blood banks, proposing confirmatory testing for those seropositive donors in screening assays and the need for adequate counseling and patient management for those confirmed HTLV infected individuals. We recommend implementing confirmatory tests at the blood bank or centralizing them within the public health system. Additionally, there is a need for a systematic approach to referring seropositive individuals to medical care, as access can be challenging.

## Materials and methods

### Ethics statement

This study was approved by the Ethical Committee on Human Research of the Universidade Federal de Mato Grosso do Sul (CEP/UFMS) by protocol number CAAE: 50,022,721.6.0000.0021. All subjects or their legal guardians, in the case of individuals under age 18, gave their written informed consent to participate in the study at the time of sampling. All research was performed following relevant guidelines and regulations.

### Study population

A cross-sectional study was conducted among blood donors from Mato Grosso do Sul state (MS)—Central Brazil, between January 2021 to December 2021. Firstly, the data was obtained from databases, and samples from the blood collection from Mato Grosso do Sul Centro de Hematologia e Hemoterapia (HEMOSUL), which has 12 units in 10 cities (Campo Grande, Dourados, Ponta Porã, Três Lagoas, Paranaíba, Coxim, Corumbá, Nova Andradina, Naviraí, and Aquidauana). The inclusion criteria were to be a blood donor in the follow-up year. Then, the screened anti-HTLV seropositive individuals were invited to participate in the study with their family members to confirm the anti-HTLV seropositivity and to invite the family members of the case to participate. Inclusion criteria were: (i) a screening diagnosis for HTLV-1 infection; (ii) being a blood donor´s family member; (iii) having signed the informed consent form.

Participants underwent an interview with a standardized questionnaire containing sociodemographic and sexual behavior information and provide blood samples. All subjects gave their written informed consent to participate in the study. In the case of individuals under age 18, informed written consent was obtained from themselves and their legal guardians.

### Serological tests

In the follow-up year, the coordinating center of HEMOSUL screened all serum samples using chemiluminescent microparticle immunoassay (CMIA) (Abbott Architect rHTLV I/II kit and ARCHITECT *i*2000SR equipment) for the presence of anti-HTLV-1/2 antibodies. It was considered positive when samples had a cut-off ≥ 1, following the manufacturer’s instructions. The grey zone considering a cut-off ranging from 0.9 to 1.0, is configured in the equipment by HEMOSUL. Positive samples were confirmed by HTLV-1/2 INNO-LIA assay (Fujirebio INNO-LIA HTLV I/II Score).

Our group retested all the positive serum samples, provided by HEMOSUL, using an enzyme-linked immunosorbent assay (ELISA) commercial kit for the presence of anti-HTLV-1/2 antibodies (Murex HTLV I + II—DiaSorin), following the manufacturer’s instructions. Positive samples were repeatedly tested and confirmed by HTLV-1/2 Western Blot (WB) assay (MP Diagnostics HTLV BLOT 2.4—Singapore). HTLV infection was defined as a repeatedly positive ELISA and positive WB (Fig. 1).

### Data analysis

All data obtained from HEMOSUL were available on the blood bank system, named HEMOVIDA, which contains information including age, sex, ethnicity, marital status, geographical origin, and the results of serological tests. Also, the questionnaire applied for the participants who consented to participate in the study containing sociodemographic and sexual behavior information.

The variables were analyzed using Stata 13.0 software (Stata Corporation, College Station, TX, USA). Categorical variables were presented with absolute and percentage frequency. Continuous variables were expressed as mean, standard deviation, median, and range.

## Data Availability

All the relevant and original data presented in the study are included in the article.
